# The Relationship between Urban Population Density Distribution and Land Use in Guangzhou, China: A Spatial Spillover Perspective

**DOI:** 10.3390/ijerph182212160

**Published:** 2021-11-19

**Authors:** Yisheng Peng, Jiahui Liu, Tianyao Zhang, Xiangyang Li

**Affiliations:** 1 School of Urban Design, Wuhan University, Wuhan 430072, China; yisheng.peng@whu.edu.cn; 2 Faculty of Construction and Environment, The Hong Kong Polytechnic University, Hongkong 999077, China; 3 Harbin Institute of Technology Shenzhen, School of Architecture, Shenzhen 518055, China; zhangtianyao@hit.edu.cn; 4 Institute of Central China Development, Wuhan University, Wuhan 430072, China; lixiangyang@whu.edu.cn

**Keywords:** urban population density distribution, land use, spatial spillover effect, spatial-temporal characteristics, Baidu heat map

## Abstract

Urban population density distribution contributes towards a deeper understanding of peoples’ activities patterns and urban vibrancy. The associations between the distribution of urban population density and land use are crucial to improve urban spatial structure. Despite numerous studies on population density distribution and land use, the significance of spatial dependence has attained less attention. Based on the Baidu heat map data and points of interests data in the main urban zone of Guangzhou, China, the current paper first investigated the spatial evolution and temporal distribution characteristics of urban population density and examined the spatial spillover influence of land use on it through spatial correlation analysis methods and the spatial Durbin model. The results show that the urban population density distribution is characterized by aggregation in general and varies on weekends and weekdays. The changes in population density within a day present a trend of “rapid growth-gentle decline-rapid growth-rapid decline”. Furthermore, the spatial spillover effects of land use exist and play the same important roles in population density distribution as the direct effects. Additionally, different types of land use show diverse direct effects and spatial spillover effects at various times. These findings suggest that balancing the population density distribution should consider the indirect effect from neighboring areas, which hopefully provide implications for urban planners and policy makers in utilizing the rational allocation of public resources and regarding optimization of urban spatial structure.

## 1. Introduction

With the rapid urbanization process, urban space, constantly expanding and reconstructing, becomes more complex [1]. A series of “urban diseases” have emerged, such as environmental pollution, traffic congestion, and separation of living and working space [2,3,4]. These issues drive changes in the dynamic distribution of urban population density which is a major perspective of the improvement on urban liability [5]. Examining population agglomeration can provide information about the aggregation pattern of residents’ behavior [2,6,7]. In addition, the distribution characteristics of urban population density reflect urban vibrancy, which is associated with urban attraction and development [8] and is significant for the allocation of public services facilities [9,10]. Therefore, it is necessary to examine the spatial and temporal dynamics of urban population density.

Recently, many scholars have stated that land use drives the dynamic distribution of urban population density [11,12,13]. Different combinations of land use have various levels of attraction to residents’ activity. Although many studies focus on improving the urban vibrancy through studying land use, the intricate relationship between them has not yet been fully and clearly clarified due to a lack of effective data and means. Traditional data like census data and survey data cannot capture the latest development trends of the spatiotemporal characteristic of population distribution and land use [14]. There is a trend in the current research of being more refined in land use classification and scale selection [8]. With the aid of information and communication technologies, the increasing interest in developing various methods from different perspectives is helpful to understand the roles of land use [15]. The Baidu heat map data and points of interest (POIs) data referring to the geographical entities are widely used to evaluate the spatiotemporal relationship of the urban land use and urban vibrancy due to the advantage of their precise spatial and temporal information [8,14,15,16]. Methodologically, multiple models had been developed to reveal the relationship between the dynamic distribution of urban population density and land use, such as the spatial Lag models (SLMs) [17], geographically weighted regression (GWR) model [18], and the geographically and temporally weighted regression (GTWR) model [8,14,15]. However, most existing research focuses on the impact of space and the spatial heterogeneity on the relationship between land use and population density; few studies take spatial correlation into consideration. Spatial correlation refers to the fact that people tend to choose neighboring zones with similar activities rather than other zones [19]. It is worth noting that it has been proven that the spatial autocorrelation of population density exists in adjacent areas [20]. Additionally, the spatial linkage of land use can efficiently relieve population pressure and realize sustainable urban development [14,21]. Rational unitization of the spatial correlation in terms of the spillover effect is beneficial to improve the efficiency of resource usage [21]. Thus, it is of great significance to study the endogenous and exogenous effects between land use and population density distribution. Furthermore, considering the dependence between spatial units can provide guidance for urban planning. To fill this research gap, this paper attempts to explore the relationship between land use and urban population density distribution from a spatial correlation perspective.

Accordingly, this study aims to explore the spatial spillover effect of land use on urban population density distribution. First, we calculated the population density index (PDI) of 7:00–24:00 on weekends and the workdays. Then, we evaluated the spatial-temporal distribution of the population density. Finally, we examined the spatial spillover effect of the relationship between land use and the distribution pattern of population density. To sum up, the paper is based on the following two aspects: (1) analyzing the temporal evolution and spatial distribution characteristics of the urban population density, and (2) investigating the spatial spillover effect of land use on urban population density distribution. Therefore, this study provides a new perspective for the population aggregation: the spatial spillover effect of land use on the population density distribution. Theoretically, we are contributing to the literature on behavior travel and urban vibrancy by putting forward suggestions for improving land use efficiency and achieving sustainable urban development.

The rest of the paper is organized as follows. Section 2 summarizes the relevant progress in the study of urban residents’ spatial–temporal activities. Section 3 is concerned with methodology and data. Section 4 analyzes the temporal evolution and spatial distribution patterns of the population density via the spatial correlation analysis method. Section 5 explores the relationship between population density and POIs via the spatial Durbin model and discusses the spatial spillover effect, while conclusions and limitations are presented in Section 6.

## 2. Literature Review

### 2.1. Urban Population Density Distribution

Urban population density distribution represents the spatial structure of population in the city [3]. Residents are more likely to travel to different areas in order to satisfy their needs for various activities [11], which results in the spatiotemporal evolution of the urban population density [11]. Meanwhile, it is worth noting that the characteristics of residents’ activities, which reflect the urban spatial structure, can be revealed from the temporal–spatial characteristics of the urban population density [22,23]. Put simply, residents’ behaviors can be predicted in time and space [24]. It is thus important to analyze the spatial–temporal distribution of urban population density to gain insight into residents’ behavior patterns and then perform rational urban planning for land use to strengthen the control of the urban population in order to alleviate problems caused by rapid urbanization [3]. Hence, research on the spatial–temporal characteristics of population density is of great significance and has received increasing attention [1,11]. However, the issue of how to quantify population density distribution first needs to be addressed.

In the past, the household survey, which is slow to update, time-consuming, and high-cost, was the major approach to investigate the distribution patterns of population density [23,25]. Fortunately, the rapid development of information and communication technologies, such as the Baidu heat map, social media data, bus smart card data, and mobile phone data, has filled the gaps of traditional data sources and provided a brand new approach for understanding urban residents’ spatial–temporal behaviors [23,26,27]. As one of the most commonly used types of geo-tagged data, the Baidu heat map can be used for spatial–temporal information [28]. Relying on the technology of Location-Based Services (LBS), the Baidu heat map records the location data of application users every 15 min, displays this location information on a map [29], and reflects population aggregation with different colors and brightness, which is the main source of exploring the spatial–temporal dynamic distribution of the urban population [11,12,13]. Thus, the Baidu heat map has the potential to deliver reliable information regarding residents’ behaviors [30,31,32]. Therefore, this study adopts the Baidu heat map to quantify population density distribution.

The distribution characteristics of population density vary at different times [33,34]. In addition, the spatial–temporal patterns of residents’ activities are not the same in different regions but are under some common laws. Within a day, the types of residents’ behaviors are dynamic and influenced by time [11]. For instance, most people are at their work place in the morning and are at home at night [35]. Furthermore, compared to working days, the duration of population density on the off-days is hysteretic, and the center of gravity of the population distribution shifts due to different activity purposes [28,34]. For example, people prefer to gather together in places of entertainment and residence at noon on rest days or after work [35]. Accordingly, location and time should be taken into consideration for studying the dynamic distribution of populations [15].

### 2.2. Population Density Distribution and Land Use

According to previous studies, land use plays an important role in urban population density distribution. Reasonable land use is more likely to balance the population density distribution. Land use can be well represented and identified with points of interest (POIs), which contain rich geospatial information, representing geographic entities [11,36,37]. On this basis, existing research has found that the land use mix—the POI variables of commerce, food, transportation, working, and housing—affect residents’ behaviors [8,38,39]. In general, urban residents are more likely to gather in areas with a high degree of land mixing [13]. The high richness of POIs refers to a combination of land use categories, which offers more attraction to urban residents [15]. In addition, different types of land use have different impacts on residents’ activities [11,15]. As Zhang et al. [8] concluded, the food, housing, and company POIs showed positive attraction to gather, while tourism POIs scattered crowds. Moreover, the influence of land use on population density distribution also varies over time [11,18,40]. It is mainly influenced by educational and business activities in the daytime, while by residence and business at night [11]. Compared to working days, residents spend more time on commercial and leisure activities [11]. Although it has been evidenced that different land use plays various roles in population density distribution at different times, the existing research ignores the interaction between land use and population density distribution.

According to the first law of geography, everything is related to everything else, but near things are more related than distant things [41]. It has been acknowledged that both land use development and population are strong external characteristics [42]. Therefore, it is necessary to explore the spatial dependence relationship between land use and population change. Generally, the existing research on the spatial effects of population distribution is based on the spatial scale above the county level. On one hand, it has been evidenced that population changes at various spatial scales are spatially dependent [43]. Urban population density on a local scale is inclined to affect the neighbor population density [44]. On the other hand, it has been proven that land use tends to affect the population growth of local and neighboring areas [44]. In addition, there is a spatial interaction between population density and land use [42]. Stronger levels of land development attract more people to the local areas and reduce the population of the adjacent areas [42,44]. Although the existing research is taken on a large scale, population density changes occur inter- or intra-area, at local and regional scales [42]. Therefore, whether the differences in land use lead to residents’ agglomeration and the spatial spillover effect on the surrounding area needs to be discussed. The Spatial Lag Model (SLM), Spatial Error Model (SEM), and Spatial Durbin Model (SDM) are the main spatial regression methods accounting for the dependence between observations. Compared to SLM and SEM, SDM as the superior one considers the spatial lag both of the dependent variables and explanatory variables [45]. Thus, to explore the spatial spillover effect of land use, SDM, which can test the extent of the spatial spillover effect [46] and avoid the omitted variables problem [45], was employed in this current research.

Hence, in this study, we attempted to use the Baidu heat map for receiving information regarding population density distribution and obtaining POI data to characterize the land use. Moreover, we aimed to adopt SDM to explore the spatial spillover effect of land use on urban population density distribution and thus to gain deeper insight into the mechanism of land-use effects on urban population density distribution.

## 3. Methodology

### 3.1. Research Area

**Figure 1:**
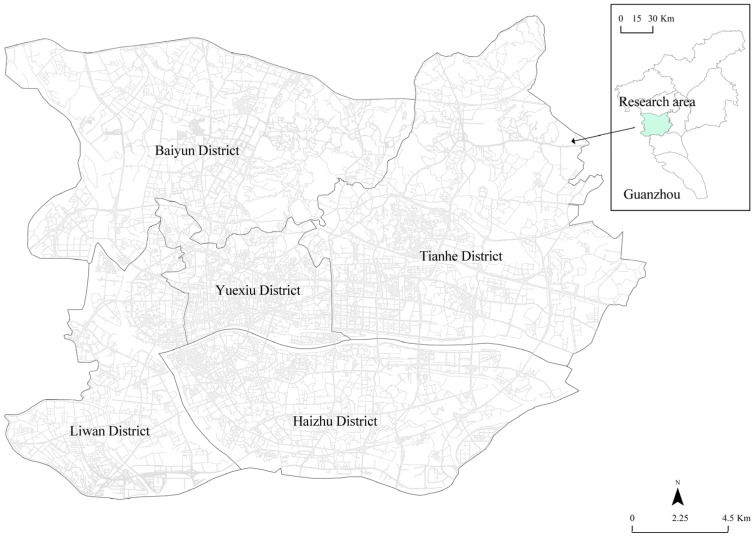
The research area.

This research area was located in Guangzhou, China (22°26′ to 23°56′ N, 112°57′ to 114°3′ E). Guangzhou, as the capital of Guangdong province, the core city of Guangdong-Hong Kong-Macao Bay area, and the hub city of the “Belt and Road”, is one of the international metropolises in China. The permanent population of Guangzhou was 1867.66 million in 2020, an increase of 47.05% over 2010. The demand for land functions increases with the increase of population, but the land use planning is inadequate, which leads to the dislocation of land use. Therefore, it is of great significance to investigate the relationship between urban population density distribution and land use. The research focuses on the main urban zone (Figure 1), about 441.71 km^2^ of Guangzhou, as the research area, involving Liwan District, Haizhu District, Yuexiu District, Tianhe District, and the southern Baiyun District (the area south of the South China Express Line).

Regarding spatial units, previous studies chose sub-districts, road network blocks, and traffic analysis zones (TAZs) as the analysis unit, which are too large to take into account the details of the characteristics of residents’ behaviors. In this study, a 500 m × 500 m grid is used as the spatial unit, to be a foundation of the analysis of the dynamic distribution of population density at a finer scale. We divided the main urban zone of Guangzhou into 1895 grids using the fishing net tool in ArcGIS10.2, then calculated population density index (PDI) by the hour for each grid and correlated the results with POI data.

### 3.2. Data Collection

The data used in this paper is Baidu heat map data and POI data.

The Baidu heat map data were collected by a plugin in ArcGIS 10.2. Based on the principle of no extreme weather, holidays, or special events, the basic data from 26 to 30 November 2020 were obtained in the research, which comprised working days and off-days. Then, we collected data once every 60 min from 7:00 to 24:00 in a single day. Finally, 90 Baidu heat maps with a spatial resolution of 3.24 m were acquired. With band 4 of the data loaded into ArcGIS10.2, the calorific value was divided into 6 categories by Natural breaks (Jenks), and then the population density and PDI of each spatial unit were calculated.

The POI dataset was obtained from the Baidu Map, one of the most popular map services in China. A total of 49,760 POIs, which included information on name, type, address, and coordinates in 14 major categories were acquired. We processed POI data using the following steps: first, the coordinates of POIs were converted from the Baidu coordinates system to WGS_1984. Second, due to the similar impact on residents’ activities, shopping POIs and leisure POIs were combined into entertainment POIs [8,12]. Similarly, commercial residence POIs and accommodation POIs were combined into housing POIs. Third, based on 4 functions of land use [27], POIs were reclassified into 12 categories, including housing POIs, life service POIs, medical and health POIs, office POIs, finance and banking POIs, government and social insurance POIs, factory POIs, transportation POIs, food POIs, entertainment POIs, education and culture POIs, and tourism POIs. Finally, land use could be well reflected by the density of POIs [11]. Hence, the density of all types of POIs was extracted in each grid in ArcGIS10.2 and standardized as shown in Table 1.

### 3.3. Analysis Framework

**Figure 2:**
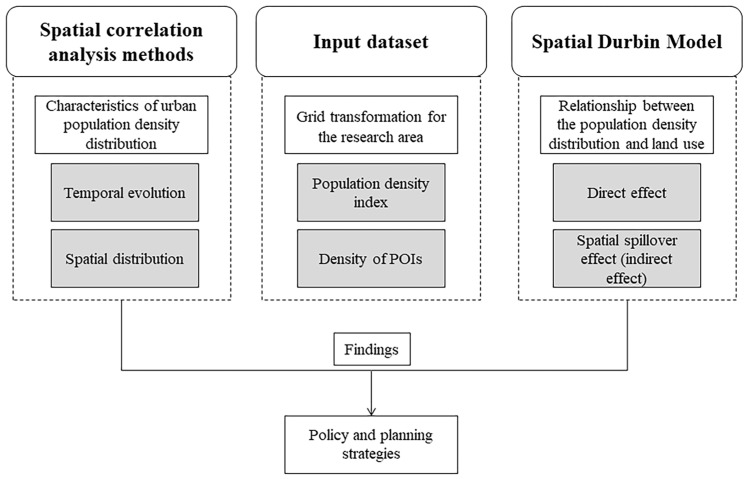
Framework for studying the urban population density distribution.

Focusing on the exploration of the spatial spillover effect of land use on the urban population density distribution, this article put forward an analysis framework to study the temporal evolution and spatial distribution of population density in terms of multilevel social sensing. As shown in Figure 2, the framework illustrated the investigation between the dynamic distribution of urban population density and land use. First, the population density index (PDI), counted from the Baidu heat map data of whole day on the weekend and on weekdays, was conducted to explore the spatial–temporal distribution characteristics of population density using spatial correlation analysis methods. Subsequently, taking the spatial spillover effect into consideration, we attempted to utilize the spatial Durbin model in exploring the correlation between the dynamic distribution of population density and land use. The details of the methods are presented in the following sections.

#### 3.3.1. Population Density Index (PDI)

The PDI, proposed by Leng et al. [47] and improved by Li et al. [12], was used to measure and forecast the urban population density distribution under the support of the Baidu heat map. The calculation formula is shown as Equation (1).

(1) $$Q=\frac{\sum_{i=1}^{m}a_{i}{\times b}_{i}\times c}{S}\;$$

where *Q* is the urban population density of a grid at a certain time, m indicates the total categories of colors we classified above, *a_i_* denotes the population density of color *i*, *b_i_* is the number of pixels of color *i*, *c* indicates the area size of a unit pixel, and *S* represents the area of each grid.

The Baidu heat map relies on users to use Baidu’s products to obtain user location information, which may lead to biases such as the fluctuation in the number of users due to users moving continuously online and offline, and the difference in preferences caused by different backgrounds [47]. To eliminate this effect, the PDI is introduced.

(2) $$\mathrm{PDI}=\frac{Q_{th}}{\sum^{}Q_{th}}$$

where t represents the time, h indicates the grid, *Q_th_* denotes the population density of the grid *h* at the time *t*, and *∑Q_th_* denotes the population density of all grids at the time *t*.

#### 3.3.2. Spatial Correlation Analysis Methods

Spatial correlation analysis methods can be used to investigate the temporal evolution and spatial distribution characteristics of population density [11,48]. Getis-Ord General G, and Getis-Ord Gi*, two spatial correlation analysis methods, are suitable to explore population agglomeration of the Baidu heat map calculated in Arcgis10.2 [12]. Getis-Ord General G is used to discuss the global correlation characteristics of population agglomeration and discover the spatial pattern of population density distribution in the whole research area. The higher (or lower) the Z score, the higher the degree of clustering, but when the Z score is 0, there is no significant clustering. Moreover, a positive Z score indicates a high-value cluster, while a negative Z score indicates a low-value cluster [12]. The Getis-Ord General G expression is shown as follows:

(3) $$G=\frac{\sum_{i=1}^{n}\sum_{j=1}^{n}W_{i,j}x_{i}x_{j}}{\sum_{i=1}^{n}\sum_{j=1}^{n}x_{i}x_{j}},\;\forall \;j\;\neq \;i\;$$

where *x_i_* and *x_j_* are the population density index of the grid *i* and *j*, respectively, *W_ij_* is the spatial weight between the grid *i* and *j*, n is the number of grids in the dataset, and ∀ *j* ≠ *i*.

The Z score is computed as

(4) $$Z=\frac{G-E\left(G\right)}{\sqrt{V\left(G\right)}}\;$$

where

(5) $$E\left(G\right)=\frac{\sum_{i=1}^{n}\sum_{j=1}^{n}W_{i,j}}{n\left(n-1\right)},\;\forall \;j\;\neq \;i\;$$

(6) $$V\left(G\right)=E\left(G^{2}\right)-E{\left(G\right)}^{2}$$

Getis-Ord Gi* is used to discuss the local correlation characteristic of population agglomeration and discover the distribution of hot spots and cold spots. If Z(Gi*) is positive and significant, it is defined as hot spots, indicating that the value around position *i* is relatively high (higher than mean). Otherwise, it is defined as cold spots, indicating that the value around position *i* is relatively low (lower than mean).

The Getis-Ord Gi* expression is shown as follows:

(7) $$Z\left(G^{*}\right)=\frac{W_{i,j}\left(d\right)x_{j}}{\sum_{j}^{n}x_{j}}$$

Gi*(d) standardizing:

(8) $$Z\left(G^{*}\right)=\frac{G_{i}^{*}-E\left(G\right)}{\sqrt{\mathrm{VAR}\left(G_{i}^{*}\right)}}$$

where E(Gi*) and VAR(Gi*) are the mathematical expectation and variance of Gi*, respectively.

#### 3.3.3. Spatial Durbin Model (SDM)

In this study, the SDM is used to examine the relationship between the urban population density distribution and land use, considering the spatial spillover effect. The SDM is one of the spatial econometric models. The other common spatial econometric models are the Spatial Lag Model (SLM) and Spatial Error Model (SEM). They are all transformed from the general spatial econometric model. The form of the general spatial econometric model is shown as follows [49]:

(9) Y=ρWY+Xβ+θWX+υ

(10) $${\upsilon =\lambda W}_{u}+\varepsilon$$

where *Y* is the explained variables, *X* is explanatory variables, *W* is the spatial weight, ρ represents the influence of *WY* in neighboring grids on the *Y*, *θ* denotes the influence of *WX* in neighboring grids on the *Y*, *β* refers to the coefficient, *λ* is the spatial autocorrelation coefficient, *W_u_* represents interaction between disturbance items of different units, and *ε* is disturbance error.

If the parameter *θ* = 0 and *λ* = 0, the SLM is defined as Equation (11). SLM is mainly used to study the interaction between explained variables.

(11) Y=ρWY+Xβ+ε

If the parameter *θ* = 0 and *ρ* = 0, the general spatial econometric model turns into the SEM, as follows. The SEM takes the interaction effect of the disturbance item into account,

(12) Y=Xβ+υ

(13) $${\upsilon =\lambda W}_{u}+\varepsilon$$

If the parameter *λ* = 0, the spatial Durbin model is defined as follows. Different from SLM and SEM, SDM not only considers the spatial correlation of the explained variables but also considers the spatial correlation of the explanatory variables [50]. Moreover, the coefficients estimated by the SDM model are decomposed into the direct effect and spatial spillover effect using the partial differential method.

(14) Y=ρWY+Xβ+θWX+ε

In the study, SDM was better suited to explore the relationship compared with OLS, SLM, and SEM. The SDM was applied in Rstudio (version 1.2) and MatLab (version R2016a). First, the “Queen” spatial weight matrix was formulated in the study. Second, with reference to previous studies [9], the data of urban population density from 7:00–24:00 in each grid were divided into 4 time periods: morning (7:00–12:00), afternoon (13:00–18:00), evening (19:00–22:00) and night (22:00–24:00). Finally, the PDI was used as the dependent variable and the 12 types of independent variables as independent variables to construct SDM.

## 4. Results

### 4.1. The Temporal Evolution Characteristics of Urban Population Density

**Figure 3:**
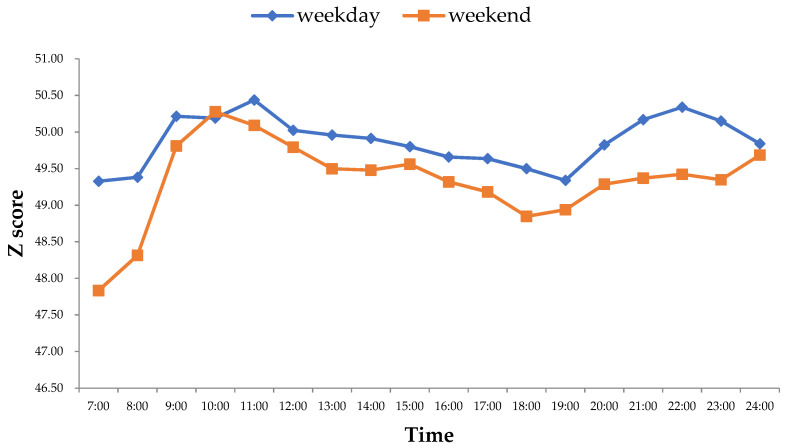
The changes of PDI of each grid on weekdays and the weekend.

The population density that presented an intensity of activity has significant differences according to time (Figure 3). In addition, the high-density population has a tendency to gather, whether it is on a weekday or the weekend. The population density of the main urban area of Guangzhou on the off-days and working days was similar and presented a trend of “rapid growth-gentle decline-rapid growth-rapid decline”. However, it is worth noting that the temporal evolution characteristics of population density were different in detail on the weekend and workdays.

On weekdays, the intensity of residents’ activities at 7:00 was the lowest in a single day. By 7:00–11:00, the intensity of residents’ activities increased rapidly and reached a peak, signaling the main gathering time for morning commuting. PDI gradually declined, while it began to rise around 19:00 and reached the second peak at 22:00. After this time, the degree of population density concentration continued to decrease. On the off-days, the population density fluctuated more drastically, and the Z score was generally lower than that on weekdays. The intensity of residents’ activities at 7:00 was also the lowest in a single day. By around 10:00, it reached the peak, which was then followed by a steady downward trend approaching 18:00. After this time, the activity intensity continued to increase gradually. Compared with that on working days, the intensity was lower on non-working days. In addition, the Z score value of PDI was smaller, indicating that the crowd tended to gather with relatively low intensity in more centers.

### 4.2. The Spatial Distribution Characteristics of Urban Population Density

**Figure 4:**
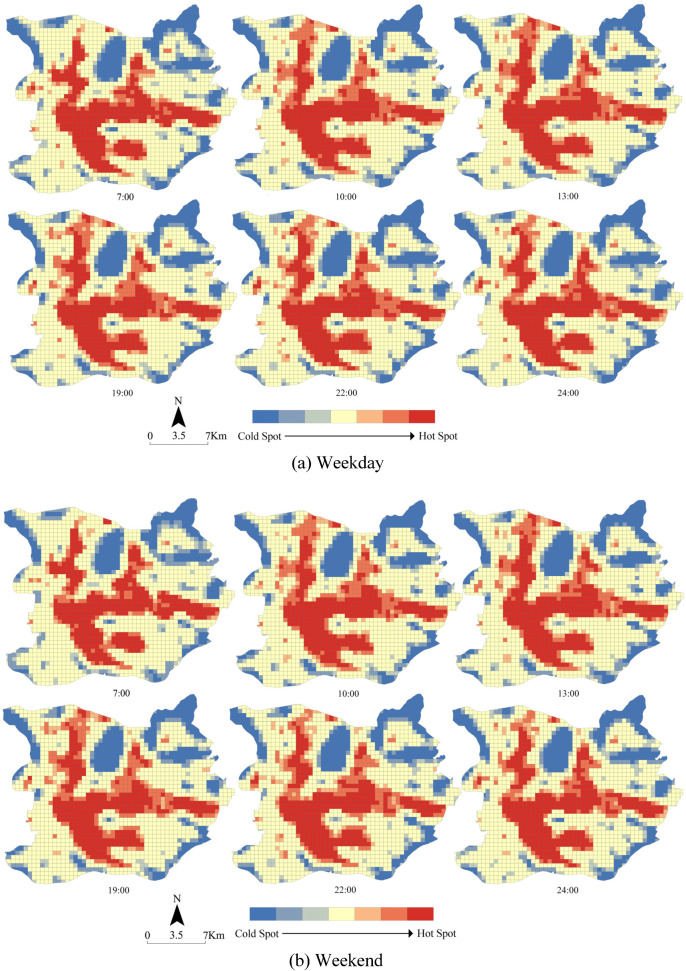
The spatial distribution of PDI on weekdays and the weekend.

Urban population density distribution, from the spatial dimension, showed obvious clustering characteristics (Figure 4). Geographically, the hot spots that denoted a high population agglomeration area covered the northeast of the Liwan District (Hualin, Longjin, Fengyuan sub-district, etc.), west of the Haizhu District (Jiangnanzhong, Changgang, Haidong sub-district, etc.), most of the Yuexiu District (Beijing, Huanghuagang, Nonglin sub-district, etc.), south of the Tianhe District (Shipai, Liede, Linhe sub-district, etc.), and the areas on both sides of Baiyun Mountain, which are located in south of the Baiyun District (Jingxi, Sanyuanli, Tangjing sub-district, etc.), indicating that the urban spatial structure showed the characteristics of polycentricity. Moreover, there is a slight difference in the spatial distribution of population density between rest days and working days. In particular, the distribution of the hot spots on non-working days was relatively sparse overall compared with that on working days.

### 4.3. The Relationship between Urban Population Density and Land Use

The results of the LM test, Wald test, and LR test are shown in Table 2. In addition, the VIF parameter is no more than seven for all independent variables in the eight qualified models. Moreover, the LM and the robust LM test for the eight models were of significance, which meant that the spatial models such as SLM, SEM, and SDM were better than the OLS model to describe the correlation [51]. The Wald test and LR test for the eight models were significant, which showed that the SDM was more appropriate in this study [50]. The SDM was built to consider endogenous interaction effects and exogenous interaction effects on the association between urban population density and land use in four time periods (Table 2). The R2 value, AIC (Akaike information criterion), and likelihood values of the SDM in each slot proved that the SDM outperforms other models in this study.

As shown in Table 3, the spatial autocorrelation coefficients (rho) of the eight spatial Durbin models are all greater than 0.69 and passed the 1% significance test. This showed that urban population density distribution in the central urban area of Guangzhou has obvious spatial dependence. That is, the population density in the grid was affected, to a certain extent, by the population density in the grids that were neighboring and similar. At the same time, the regression coefficients of all types of land use except TO were positive, which explains that those factors have an obvious promotion function for population density distribution. However, the regression coefficients of the spatial lag of variables would affect the feedback effect [52]; it was thus necessary to decompose the spatial effect into direct effect and spatial spillover effect.

Moreover, the direct effect and spatial spillover effect of explanatory variables were solved by the partial differential decomposition method, and the results are shown in Table 4. The direct effect, also named the local effect, represents the influence of the land use types of the grid on the population aggregation of the grid, while the spatial spillover effect, also named the indirect effect, represents the impact of the local grid land use types on the grid population agglomeration of neighboring grids.

For the direct effects, the influencing coefficients of four functions of land use on urban population density distribution varied within a day, and the intensity between off-days and working days was also different. First, the direct effect coefficients of HP, LS, and MH on residents’ behaviors were positive. The trends of living function were similar throughout the day, whether it was on the working days or off-days. Specifically, the trends formed a trough in the afternoon and then rose, showing a “V” shape in a single day, which was in line with people’s living habits. It is noteworthy that the direct effect coefficients of LS in the morning and night were not significant, which may be related to residents’ activity habits and the bias of the Baidu heat map data [11]. Meanwhile, the degree of impact of HP and LS on the off-days was higher than that on the weekdays, but the degree of impact of MH on the off-days was lower than that on the weekdays. In terms of working function, OF, GS, and FA had a significant positive relationship to urban population density distribution. Furthermore, from the perspective of influencing intensity, OF, GS, and FA were lower on the off-days than that on the working days, which would be expected. In terms of transportation function, the direct effect coefficients of TR on residents’ activities were positive. Whether the trend was on the weekend or weekdays, it presented a pattern of “growth-decrease”, with the peak appearing in the afternoon throughout the day. Moreover, the intensity of the non-working days was lower than that of the working days in the morning and afternoon, while in the evening and night, the intensity of the non-working days was higher. In terms of recreation function, the direct effect coefficients of FO, EN, and EC were positive, while the direct effect coefficients of TO were negative. From the perspective of influencing intensity, EN and TO on the off-days were higher than on the working days, and EC on the off-days was lower than that on the weekdays. Interestingly, FO on the working days was lower in the morning and afternoon than on the rest days, and lower in the evening than on the rest days.

For spatial spillover effects, 9 of the 12 independent variables had a significant relationship with population density distribution, showing the siphoning effect and the trickle-down effect of land use on the population agglomeration in the surrounding area. In terms of living function, HO had a negative effect on residents’ behaviors, while LS and MH had positive effects on it, indicating that HO would reduce the population density in the surrounding area while LS and MH would drive an increase in population density. In terms of working function, FB had a negative indirect effect on residents’ behaviors, while OF and FA had positive effects on it, which denoted that the more FB in the grid, the less the population density nearby, while OF and FA would not increase population density nearby. In terms of transportation function, TR had a positive spatial spillover effect on population agglomeration, indicating that the increase of transportation facilities in the target grids would lead to the increment of population density in the neighboring grids. In terms of recreation function, the spillover effect coefficients of FO and EC were positive.

## 5. Discussion

The spatial–temporal dynamic distribution of urban population density is one of the manifestations of urban vibrancy and also a demonstration of the match between residents’ activities and urban spatial functions.

### 5.1. Characteristics of Urban Population Density Distribution

The spatial–temporal evolution of urban population density distribution showed the difference between working days and non-working days. From the perspective of temporal evolution, the degree of crowd agglomeration on the weekdays was greater than that on the weekend, which was in line with the conclusions drawn from other research areas [11,12,13]. Additionally, the fluctuation range on non-working days was at a higher level. However, this finding was not exactly the same with other research. Feng et al. evidenced that the fluctuation of population density distribution on weekdays was higher than that on the weekend [12]. From the perspective of spatial distribution, the crowd was highly concentrated at a few centers on the weekdays, while it was relatively scattered in different locations on the weekend. In Guangzhou, working space was mainly concentrated in the center. In contrast, the residential distribution showed a trend of suburbanization and was scattered [16,53,54]. On the contrary, this result was not in line with the previous studies, which took other regions as the study area. Wu and Ye found that the crowd distribution was more dispersed on weekdays than on weekends from the perspective of space in the central city of Shanghai [28]. Coincidentally, hot spots in Shehong County overlapped highly on the weekdays and the weekend, which was proven by Feng et al. [12].

In summary, in addition to the errors caused by the modifiable areal unit problem (MAUP) and index selection [12,55], different land use between regions would also lead to different population distribution characteristics [3,13,31]. It was proven that land use had an important impact on population aggregation and was the key to understand residents’ activities.

### 5.2. The Impact Mechanism of Urban Population Density Distribution

Through spatial correlation analysis, the results presented a clear spatial association that existed among the population density distribution. To further analyze the mechanisms of impact on population density distribution, the direct and spatial spillover effects of land use affecting residents’ activities in the SDM model were analyzed, which provided the theoretical bases for the hypothesis of the influence of land use on population agglomeration. 

In this study, the land use of living function, on the whole, had significant positive direct effects on residents’ activities. For HO and LS, the degree of direct influence was lowest in the afternoon. The intensity of direct effects on the weekdays was lower than that on the weekend, which illustrated that residents were more willing to stay at home on non-working days, as reported in a previous study [8]. Inconsistently, the degree of MH was greater than that on the weekend. This finding on MH was in agreement with the finding of Zhang et al. [8] and Li et al. [56]. The reason may be that the number of doctors who were on duty on the weekend was less than on the weekdays due to the holidays in China, which led to a decrease in patients. Moreover, in terms of spatial spillover effects, living function locations showed different indirect effects on population aggregation. The increase of MH and LS in the local area caused the growth of the population density of neighboring areas due to the trickle-down effect, while the increase of HO in the local area had a siphonic effect on the increase of the population density of the neighboring areas. Generally, in Guangzhou, residential areas are mainly distributed along traffic routes [57]. Additionally, due to the characteristics of scarcity, high concentration, and irreplaceability of medical facilities, residents have to gather in some areas where high-quality medical institutes are located, which promotes population density in the target areas and neighboring areas [58]. 

Among the four categories of working function, OF, GS, and FA were found to have positive direct associations with population agglomeration. The trends of OF, GS, and FA in a day were various, and the intensity of direct effects on the weekdays was lower than that on the weekend. Similar findings were found in previous studies [8,13]. It was generally recognized that residents usually went to work from Monday to Friday and took a break on Saturday and Sunday. In terms of spatial spillover effects, OF, FB, and FA had a significant relationship with population density distribution. Unlike that of OF and FA, the effect of FB was negative in the main urban area of Guangzhou. Finance and banking facilities in the main urban area of Guangzhou were mainly located in the center, where crowds were not concentrated, especially in major business districts such as Zhujiang new town and Taojin [57,59]. Hence, FB had strong negative spatial spillover effects on residents’ behaviors.

The results showed land use of transportation function was the most significant factor that influences urban population density distribution. The SDM showed that the direct effects and the indirect effects of TR were more than that of other functions, presenting that residents’ activities relied on traffic accessibility, which played an important role in population aggregation [15,35,60]. The trends of direct coefficients in a day were in line with the features of commuting on the weekdays [15]. In addition, the effects on the weekdays were generally lower than that on the rest days in the afternoon and night. The result denoted that people prefer resting on non-working days, and their time for activities is delayed, as reported in a previous study [11]. 

Land use of recreation function was relevant to the urban population density distribution, which was consistent with previous studies [8,11,60,61]. For FO and EN, the positive direct influences on the off days were greater than on the working days at a certain time, displaying that FO was more attractive to residents in the afternoon and evening on the weekend, while EN was most attractive to residents in all time slots on weekdays. FO had a significant spatial spillover effect on residents’ behaviors; on the contrary, EN did not. As we know, Guangzhou is the capital of food. In other words, Guangzhou is known for its diverse food culture [8]. Hence, FO had more attraction to tourists and locals and brought population density growth to the neighboring areas, compared to EN. For EC, it had the direct and spatial spillover effects on population density distribution. This implied that, on the one hand, the urban population density distribution was mainly influenced by schooling activities, which is similar to the descriptions of Li et.al. [11]; on the other hand, due to the compulsory Education Enrollment Policy in China, residents who had children of school age gathered around the EC, especially high-quality schools, showing that EC has a siphonic effect on the increase of the population density of the surrounding areas. For TO, the significant direct effects on the non-working days were higher than that on the working days. Peoples’ activities focused on recreation and living, and TO was a good choice during leisure time.

## 6. Conclusions

In this study, we first analyzed the spatial–temporal evolution of urban population density in the main urban area of Guangzhou and then explored the direct and spatial spillover effects on land use using Baidu heat map data and POI data. The findings from our study can provide help for urban planners and policy makers in understanding residents’ activity patterns and addressing the problem about overcrowding and chaos in urban spatial structure.

Based on the above analysis of population density distribution, four suggestions are provided. First, according to the difference in peoples’ activities on the weekend and the weekdays, a flexible mechanism for urban management could be established to relieve the pressure during journey peaks. Second, on the basis of the peoples’ activities patterns, land use structure should be optimized in order to form a polycentric urban pattern and disperse population density. Third, considering spatial correlation and spillover effects, planners should coordinate land use layout with surrounding areas to improve the well-being of residents. Fourth, the principles of fairness and difference should be taken into account within the main urban area of Guangzhou. This requires the equal arrangement of high-quality infrastructure on one hand and the discovery of regional characteristics and uniqueness on the other.

In the exploration of the purpose of human activities, this current research took the spillover effect of urban population density distribution into consideration using the SDM, which made it easier to observe the influence of spatial correlation. However, there are some limitations of this study, which need to be addressed to carry out further research. First, Baidu heat map data were used as the basis for urban population density distribution and could not be used to obtain accurate population numbers or individual information. Moreover, the Baidu heat map data were collected over five days in November, which could not reflect urban population density distribution all year. Therefore, this paper needs to integrate other data that contain individual information to reduce the sample bias and effectively identify residents’ activities in different groups. Furthermore, future research should combine multi-source data and machine learning, especially domain adaption techniques [62,63], to better capture the characteristics of land use and to simulate residents’ behavior and predict the population density distribution in alternative cities, which can further illuminate the relationship between them.

## Figures and Tables

**Table 1:** Classification and summary statistics of the independent variables.

Functions	POI Categories	Item Label	Mean	SD
Living	Density of housing POIs	HO	0.0407	0.0794
	Density of life service POIs	LS	0.0342	0.0693
	Density of medical and health POIs	MH	0.0322	0.0916
Working	Density of office POIs	OF	0.0679	0.1321
	Density of finance and banking POIs	FB	0.0536	0.1120
	Density of government and social insurance POIs	GS	0.0353	0.0677
	Density of factory POIs	FA	0.0196	0.0654
TransportationRecreation	Density of transportation POIs	TR	0.0208	0.0351
	Density of food POIs	FO	0.0276	0.0769
	Density of entertainment POIs	EN	0.0505	0.0772
	Density of education and culture POIs	EC	0.0263	0.0570
	Density of tourism POIs	TO	0.0217	0.0540

**Table 2:** Comparison between different models.

		Morning: 07:00 to 12:00		Afternoon: 13:00 to 18:00		Evening: 19:00 to 21:00		Night: 22:00 to 24:00	
		Weekday	Weekend	Weekday	Weekend	Weekday	Weekend	Weekday	Weekend
LM-lag		1342.1 ***	1276.6 ***	1421.3 ***	1392.3 ***	1388.6 ***	1346 ***	1267.2 ***	1225.3 ***
Robust LM-lag		286.2 ***	280.51 ***	269.97 ***	268.15 ***	276.19 ***	270.68 ***	273.68 ***	269.23 ***
LM-error		1145.7 ***	1074.6 ***	1247 ***	1209.6 ***	1196.5 ***	1150.1 ***	1071.1 ***	1026 ***
Robust LM-error		89.79 ***	78.514 ***	95.697 ***	85.456 ***	84.046 ***	74.71 ***	77.571 ***	69.966 ***
Wald spatial lag		1762.9 ***	1692.2 ***	1820.3 ***	1803.6 ***	1794.8 ***	1732.5 ***	1656.5 ***	1606.4 ***
Wald spatial error		2877.8 ***	2721.9 ***	2877.5 ***	2831.8 ***	2807.9 ***	2673 ***	2632.4 ***	2526 ***
LR spatial lag		1070.4 ***	1025.3 ***	1100.4 ***	1081.8 ***	1081.1 ***	1047.2 ***	1011.8 ***	981.44 ***
LR spatial error		997.15 ***	948.06 ***	1035.5 ***	1013.4 ***	1006.3 ***	970.11 ***	936.48 ***	903.6 ***
R^2^	OLS	0.5256	0.5251	0.4974	0.4922	0.4982	0.4928	0.5207	0.5185
	SLM	0.7550	0.7484	0.7454	0.7404	0.7431	0.7355	0.7441	0.7386
	SEM	0.7588	0.7515	0.7496	0.7438	0.7458	0.7374	0.7472	0.7410
	SDM	0.7664	0.7598	0.7558	0.7503	0.7528	0.7449	0.7557	0.7496
AIC	OLS	−8769.5	−8679.4	−8841.7	−8835.6	−8738.5	−8696.7	−8531.8	−8491.7
	SLM	−9837.9	−9702.7	−9940.1	−9915.4	−9817.6	−9741.9	−9541.6	−9471.1
	SEM	−9764.6	−9625.5	−9875.2	−9847	−9742.8	−9664.8	−9466.3	−9393.3
	SDM	−9909.2	−9775.3	−9995	−9967.9	−9871.4	−9793.4	−9614.3	−9538.7
Log-likelihood	OLS	4398.743	4353.713	4434.858	4431.803	4383.244	4362.346	4279.908	4259.834
	SLM	4933.926	4866.371	4985.034	4972.691	4923.795	4885.963	4785.787	4750.552
	SEM	4897.319	4827.744	4952.621	4938.503	4886.383	4847.403	4748.147	4711.636
	SDM	4981.591	4914.637	5024.514	5010.960	4962.715	4923.717	4834.130	4796.329

Note: *** indicate significance at the 0.1% level.

**Table 3:** The estimation results of the SDM.

	Morning: 07:00 to 12:00		Afternoon: 13:00 to 18:00		Evening: 19:00 to 21:00		Night: 22:00 to 24:00	
	Weekday	Weekend	Weekday	Weekend	Weekday	Weekend	Weekday	Weekend
Intercept	0.0039 *** (4.4414)	0.0038 *** (4.2698)	0.0041 *** (4.7476)	0.0041 *** (4.7176)	0.0039 *** (4.3495)	0.0039 *** (4.3512)	0.0034 *** (3.7476)	0.0035 *** (3.7597)
HO	0.0381 *** (4.6188)	0.0439 *** (5.1208)	0.0247 *** (3.065)	0.0251 *** (3.0965)	0.0344 *** (4.1366)	0.0362 *** (4.2512)	0.0553 *** (6.186)	0.0565 *** (6.184)
LS	0.0428 *** (6.2719)	0.0515 *** (7.2663)	0.0320 *** (4.8088)	0.0369 *** (5.507)	0.0414 *** (6.0169)	0.0427 *** (6.0836)	0.0517 *** (6.9967)	0.0552 *** (7.3077)
MH	0.0197 *** (4.3296)	0.0199 *** (4.2184)	0.0157 *** (3.5324)	0.0145 *** (3.2361)	0.0163 *** (3.5425)	0.0165 *** (3.5237)	0.0209 *** (4.2313)	0.0215 *** (4.2781)
OF	0.0141 *** (3.1298)	0.0080* (1.7166)	0.0170 *** (3.8731)	0.0086* (1.9483)	0.0104** (2.2925)	0.0068 (1.4702)	0.0061 (1.2529)	0.0048 (0.9699)
FB	0.0106 * (1.9523)	0.0106 * (1.8867)	0.0078 (1.4783)	0.0073 (1.3642)	0.0077 (1.4147)	0.0070 (1.2432)	0.0095 (1.6138)	0.0092 (1.5367)
GS	0.0288 *** (3.5038)	0.0236 *** (2.7648)	0.0244 *** (3.0544)	0.0196** (2.4249)	0.0236 *** (2.8458)	0.0223 *** (2.636)	0.0285 *** (3.2075)	0.0279 *** (3.0738)
FA	0.0266 *** (3.5558)	0.0268 *** (3.451)	0.0317 *** (4.3429)	0.0261 *** (3.5478)	0.0266 *** (3.5283)	0.0257 *** (3.3337)	0.0272 *** (3.3511)	0.0256 *** (3.0903)
TR	0.0894 *** (7.3976)	0.0829 *** (6.6063)	0.0959 *** (8.1467)	0.0914 *** (7.6993)	0.0848 *** (6.9522)	0.0868 *** (6.9653)	0.0754 *** (5.762)	0.0772 *** (5.7771)
FO	0.0177 *** (2.9185)	0.0150 ** (2.384)	0.0242 *** (4.0993)	0.0227 *** (3.8149)	0.0235 *** (3.8429)	0.0250 *** (4.0005)	0.0153 ** (2.3339)	0.0170 ** (2.5387)
EN	0.0417 *** (4.7309)	0.0544 *** (5.9455)	0.0405 *** (4.7212)	0.0518 *** (5.9811)	0.0473 *** (5.3195)	0.0514 *** (5.6646)	0.0546 *** (5.7205)	0.0556 *** (5.7031)
EC	0.0447 *** (5.353)	0.0349 *** (4.027)	0.0472 *** (5.7977)	0.0391 *** (4.767)	0.0434 *** (5.1574)	0.0392 *** (4.5613)	0.0387 *** (4.2836)	0.0356 *** (3.8562)
TO	−0.0190 ** (−2.2209)	−0.0109 (−1.235)	−0.0165 ** (−1.988)	0.0020 (0.2366)	−0.0235 *** (−2.7319)	−0.0211 ** (−2.4021)	−0.0246 *** (−2.6558)	−0.0243 ** (−2.5697)
W × HO	−0.0655 *** (−3.9644)	−0.0726 *** (−4.2364)	−0.0525 *** (−3.2625)	−0.0555 *** (−3.4188)	−0.0634 *** (−3.8061)	−0.0642 *** (−3.7736)	−0.0839 *** (−4.6881)	−0.0850 *** (−4.651)
W × LS	0.0984 *** (5.7505)	0.1014 *** (5.6856)	0.0867 *** (5.2593)	0.0837 *** (5.0275)	0.0849 *** (4.9602)	0.0872 *** (4.9839)	0.0983 *** (5.311)	0.1005 *** (5.3086)
W × MH	0.0184 (1.3642)	0.0208 (1.4848)	0.0161 (1.2236)	0.0160 (1.2073)	0.0162 (1.1908)	0.0166 (1.1948)	0.0251* (1.7155)	0.0220 (1.4689)
W × OF	0.0146 (1.4225)	0.0211 ** (1.983)	0.0091 (0.9122)	0.0157 (1.5675)	0.0143 (1.387)	0.0171 (1.6247)	0.0234 ** (2.1119)	0.0224 ** (1.9845)
W × FB	−0.0430 *** (−3.5325)	−0.0451 *** (−3.5735)	−0.0369 *** (−3.1171)	−0.0357 *** (−2.9924)	−0.0369 *** (−3.0118)	−0.0360 *** (−2.8734)	−0.0448 *** (−3.4011)	−0.0433 *** (−3.2197)
W × GS	−0.0346 * (−1.8268)	−0.0312 (−1.5847)	−0.0299 (−1.6202)	−0.0293 (−1.573)	−0.0285 (−1.4905)	−0.0294 (−1.5067)	−0.0335 (−1.6334)	−0.0318 (−1.5174)
W × FA	0.0011 (0.0886)	0.0039 (0.2951)	−0.0024 (−0.1972)	0.0016 (0.1307)	0.0035 (0.2756)	0.0048 (0.3697)	0.0089 (0.6458)	0.0108 (0.7694)
W × TR	0.0746 ** (2.1189)	0.0854 ** (2.3376)	0.0584 * (1.7022)	0.0681 ** (1.9692)	0.0913 ** (2.5692)	0.0971 *** (2.6724)	0.1046 *** (2.7457)	0.0997 ** (2.5637)
W × FO	0.0219 * (1.7473)	0.0206 (1.5859)	0.0190 (1.5551)	0.0190 (1.5468)	0.0212 * (1.6758)	0.0193 (1.4906)	0.0265 * (1.9525)	0.0256 * (1.8518)
W × EN	−0.0309 (−1.4839)	−0.0380 * (−1.7573)	−0.0318 (−1.5696)	−0.0369 * (−1.8026)	−0.0367 * (−1.7497)	−0.0406 * (−1.8931)	X0.0379 * (−1.6789)	−0.0367 (−1.591)
W × EC	0.0035 (0.2152)	0.0119 (0.7103)	−0.0049 (−0.311)	−0.0007 (−0.0431)	−0.0003 (−0.0179)	0.0020 (0.118)	0.0041 (0.2316)	0.0078 (0.439)
W × TO	0.0017 (0.0872)	−0.0048 (−0.2407)	0.0045 (0.2392)	−0.0042 (−0.2198)	0.0088 (0.4537)	0.0083 (0.4152)	0.0048 (0.2274)	0.0046 (0.2158)
rho	0.7085	0.6982	0.72675	0.7241	0.71804	0.71263	0.69603	0.69178

Note: ***, **, and * indicate significance at the 1%, 5%, and 10% levels, respectively.

**Table 4:** Direct effects and spatial spillover effects of the SDM.

**Direct Effects**								
	**Morning:** **07:00 to 12:00**		**Afternoon:** **13:00 to 18:00**		**Evening:** **19:00 to 21:00**		**Night:** **22:00 to 24:00**	
	**Weekday**	**Weekend**	**Weekday**	**Weekend**	**Weekday**	**Weekend**	**Weekday**	**Weekend**
HO	0.0316 ***	0.0370 ***	0.0184 **	0.0184 **	0.0277 ***	0.0295 ***	0.0479 ***	0.0490 ***
LS	0.0647	0.0741	0.0517 ***	0.0566 ***	0.0614 ***	0.0630 ***	0.0737	0.0775
MH	0.0252 ***	0.0257 ***	0.0206 ***	0.0192 ***	0.0211 ***	0.0214 ***	0.0274 ***	0.0275 ***
OF	0.0183 ***	0.0124 **	0.0209 ***	0.0126 ***	0.0143 ***	0.0106 **	0.0106 **	0.0090 *
FB	0.0046	0.0044	0.0022	0.0018	0.0023	0.0016	0.0032	0.0033
GS	0.0264 ***	0.0211 **	0.0223 **	0.0169 *	0.0215 **	0.0200 **	0.0263 ***	0.0259 ***
FA	0.0300 ***	0.0305 ***	0.0354 ***	0.0297 ***	0.0306 ***	0.0297 ***	0.0317 ***	0.0302 ***
TR	0.1128 ***	0.1064 ***	0.1190	0.1153 ***	0.1114 ***	0.1141 ***	0.1011 ***	0.1019 ***
FO	0.0235 ***	0.0201 ***	0.0308 ***	0.0290 ***	0.0301 ***	0.0313 ***	0.0214 ***	0.0230 ***
EN	0.0415 ***	0.0544 ***	0.0401 ***	0.0519 ***	0.0468 ***	0.0508 ***	0.0546 ***	0.0559 ***
EC	0.0507 ***	0.0409 ***	0.0524 ***	0.0440 ***	0.0488 ***	0.0444 ***	0.0438 ***	0.0408 ***
TO	−0.0210 **	−0.0130	−0.0179 **	0.0015	−0.0249 ***	−0.0223 **	−0.0266 ***	−0.0262 ***
**Spatial Spillover Effects**								
	**Morning:** **07:00 to 12:00**		**Afternoon:** **13:00 to 18:00**		**Evening:** **19:00 to 21:00**		**Night:** **22:00 to 24:00**	
	**Weekday**	**Weekend**	**Weekday**	**Weekend**	**Weekday**	**Weekend**	**Weekday**	**Weekend**
HO	−0.1255 **	−0.1323 ***	−0.1205 **	−0.1286 **	−0.1305 **	−0.1273 **	−0.1419 ***	−0.1416 ***
LS	0.4196 ***	0.4325 ***	0.3826 ***	0.3802 ***	0.3863 ***	0.3891 ***	0.4200 ***	0.4275 ***
MH	0.1057 **	0.1094 **	0.0957 **	0.0912 *	0.0940 **	0.0940 *	0.1240 ***	0.1137 **
OF	0.0802 **	0.0840 ***	0.0748 **	0.0758 **	0.0735 **	0.0727 **	0.0865 ***	0.0794 **
FB	−0.1156 ***	−0.1187 ***	−0.1088 ***	−0.1050 ***	−0.1057 ***	−0.1027 **	−0.1195 ***	−0.1138 ***
GS	−0.0464	−0.0463	−0.0423	−0.0520	−0.0390	−0.0447	−0.0427	−0.0385
FA	0.0652 *	0.0712 **	0.0716 **	0.0707 **	0.0764 **	0.0766 **	0.0870 **	0.0880 **
TR	0.4499 ***	0.4510 ***	0.4458 ***	0.4625 ***	0.5130 ***	0.5257 ***	0.4913 ***	0.4719 ***
FO	0.1122 ***	0.0977 **	0.1273 ***	0.1222 ***	0.1283 ***	0.1225 ***	0.1160 ***	0.1153 ***
EN	−0.0044	−0.0002	−0.0082	0.0020	−0.0094	−0.0131	0.0003	0.0054
EC	0.1146 **	0.1143 **	0.1021 **	0.0951 *	0.1041 **	0.0989 **	0.0969 **	0.1001 **
TO	−0.0383	−0.0393	−0.0262	−0.0094	−0.0271	−0.0225	−0.0386	−0.0375

Note: ***, **, and * indicate significance at the 1%, 5%, and 10% levels, respectively.

## Data Availability

Data of the present study are available on request from the corresponding author.
